# PROMPT intervention for children with severe speech motor delay: a randomized control trial

**DOI:** 10.1038/s41390-020-0924-4

**Published:** 2020-05-01

**Authors:** Aravind K. Namasivayam, Anna Huynh, Francesca Granata, Vina Law, Pascal van Lieshout

**Affiliations:** 1 Oral Dynamics Laboratory, Department of Speech–Language Pathology, University of Toronto, Toronto, ON Canada; 2 Toronto Rehabilitation Institute, Toronto, ON Canada; 3 Rehabilitation Sciences Institute, University of Toronto, Toronto, ON Canada

## Abstract

**Background:**

Currently, there is limited information on the intervention efficacy for children with speech motor delay (SMD). This randomized control trial (RCT) study examined the effectiveness of Prompts for Restructuring Oral Muscular Phonetic Targets (PROMPT) intervention to improve the outcomes in children with SMD. We hypothesized that children with SMD receiving PROMPT intervention would improve more in the measured outcomes than those waitlisted and receiving home training.

**Methods:**

Using a two-arm, parallel group, RCT, 49 children with SMD were allocated to either an intervention group (*N* = 24) that received 45 min of PROMPT intervention two times a week for 10 weeks or were waitlisted for the same duration and received only home training instructions (*N* = 25). Outcome measures for speech motor control, articulation, speech intelligibility (word and sentence levels), and functional communication were assessed at baseline and at a 10-week follow-up.

**Results:**

PROMPT intervention was associated with notable improvements in speech motor control, speech articulation, and word-level speech intelligibility. Intervention allocation yielded weak improvements in sentence-level speech intelligibility and functional communication.

**Conclusions:**

PROMPT intervention is a clinically effective intervention approach for children with SMD.

**Impact:**

- Currently, there is limited information on the intervention efficacy for children with SMD.
- We report on the findings of a phase III intervention efficacy study on children with SMD using an RCT design.
- PROMPT intervention is a clinically effective intervention approach for children with SMD.
- Results of the study will be fundamental to the delivery of effective services for this population.
- These findings may facilitate the development of an evidence-based care pathway for children with severe speech sound disorders.

## Introduction

The Speech Sound Disorder Classification System^1,2^ categorizes motor-speech disorders (MSD) into four categories: childhood apraxia of speech (CAS), dysarthria (DYS), concurrent CAS and DYS, and speech motor delay (SMD; formerly known as MSD – not otherwise specified; MSD-NOS;^1,2^ also referred to as speech sound disorders with motor-speech involvement (SSD-MSI^3^) prior to 2017).

In general, children with MSD are resistant to traditional articulation and phonological intervention approaches and are at a greater risk for persistent SSD.^3–6^ As a result, these children are more likely to experience short- and long-term difficulties in social, emotional, and academic domains.^7^ To limit the impact of SSD on those domains, it is critical to identify the specific nature of a child’s speech disorder and select clinically effective interventions.

For both CAS and DYS populations, intervention efficacy has been recently demonstrated using randomized control trials (RCTs);^8,9^ however, only limited information is presently available on the nature, diagnosis, and intervention efficacy for children with SMD.^10,11^ The present study focused on children with SMD. It is estimated that between 10% and 12% of children presenting with idiopathic speech delay are clinically significant for SMD with a population-based prevalence of four children per 1000.^2,11^ Children with SMD present with a pathophysiology at the level of neuromotor execution of speech related to a limitation or delay in the development and maturation of speech motor skills required for precision and stability of speech, voice, and prosodic output.^2^ Clinically, these children may present with decreased jaw stability (e.g., lateral jaw sliding), limited control of the degree of jaw height (jaw grading) for mid-vowels (e.g., [e], [o], [ɛ], and [ɔ]), excessive jaw movement range, decreased lip rounding and retraction, and occasionally overly retracted lips.^3,5^

Intervention outcome data for this population is scarce with only two single - subject multiple probe design studies^12,13^ and one single group pre–post test design study^5^ representing phase I clinical research.^14^ These phase I studies with small sample sizes examined intervention effects, and the magnitude of these effects to determine whether the intervention is appropriate for further refinement (phase II) and subsequent clinical trials (phase III). Since then, several phase II studies have refined the definition of this population,^3,10^ identified appropriate outcome measures,^15^ replicated the magnitude of change (effect size) from phase I studies,^3^ developed standardized measurement procedures to assess intervention fidelity,^16^ and most importantly, determined optimal intervention dosage parameters^3^ for SMD.

From the sparse data that is available, interventions such as the Motor-Speech Treatment Protocol (MSTP)^3,12^ and Prompts for Restructuring Oral Muscular Phonetic Targets (PROMPT^4,5^) that integrate information across auditory, visual, and tactile–kinesthetic systems have demonstrated positive improvements in speech production and speech intelligibility for this population. Although both the MSTP^12^ and PROMPT have emerging (phase I/II) evidence for the intervention of SMD, PROMPT was selected as the intervention approach in the current study because it focuses on improving the accuracy^5,13^ as well as the stability of speech production,^4,17^ which makes it more suited to address the core issues in this population. The purpose of this paper is to report on the findings of a phase III intervention efficacy study using PROMPT on children with SMD using a randomized control trial (RCT) design. Currently, these data do not exist, and results from this study will be fundamental to informing the delivery of effective services for this population.

## Study aims and hypotheses

The study examined the effectiveness of PROMPT (10-week, 2×/week) intervention, in comparison to a waitlist/home training group to improve the outcomes in children with SMD (previously referred to as SSD-MSI or MSD-NOS^1^) using the World Health Organization’s International Classification of Functioning, Disability and Health: Child and Youth (WHO-ICF-CY^18^) framework. As per the WHO-ICF-CY framework, we assessed the following levels: (1) body structures and functions: speech motor control, speech articulation, and speech intelligibility; and (2) activities and participation level: functional communication. We hypothesized that children with SMD receiving PROMPT (10-week, 2×/week) intervention would improve outcomes, more so than those waitlisted and receiving home training.

## Methods

### Design

This study used a multi-site, two-arm, parallel group, RCT design with concealed group allocations. In this study, both the investigator and outcome assessors were blinded to the group allocation. One arm received 10 weeks of PROMPT intervention (intervention group), while the other arm was waitlisted for the same period and received routine home training instructions (waitlist/home training group). The study integrity was monitored by an arm’s-length, external agency, The Applied Health Research Center (AHRC) at St. Michael’s Hospital in Toronto, Ontario, Canada. The AHRC was responsible for verifying the consent process; ensuring participants met study inclusion/exclusion criteria; conducting on-site data monitoring visits; centrally administering randomized group allocation via sequentially numbered and opaque-sealed envelopes; verifying source data and data entry; conducting a priori and interim power analysis and all other statistical analysis on outcome measures.

### Participants and setting

Children were recruited from three community-based healthcare centers (in Mississauga, Toronto, and Windsor, Ontario, Canada). Children were eligible to participate in the study if they met the following inclusion criteria: (a) aged between 3 and 10 years of age; (b) presented with a moderate-to-severe SSD (≤64% severity determined by the percentage of consonants correct (PCC)^19^) and were classified descriptively as having SMD using features reported in the precision stability index;^1,10^ (c) have English as the primary language spoken at home; (d) hearing and vision within normal limits; (e) non-verbal intelligence at or above the 25th percentile (average/within normal limits on the cognitive test with a standard score ≥90; Primary Test of Non-verbal Intelligence (P-TONI)^20^); (f) age-appropriate or mildly delayed receptive language skills (CELF-P2: Clinical Evaluation of Language Fundamentals – Preschool 2nd edition for children between 3 and 6 years;^21^ CELF-4: Clinical Evaluation of Language Fundamentals – 4th edition for students between 5 and 21 years;^22^ standard score ≥78); (g) presence of a minimum of four out of nine indicators for MSI (1. lateral jaw sliding, 2. decreased lip rounding and retraction, 3. inadequate integration of jaw and lips across two planes of movement (front/back and up/down, such as needed in the sounds produced in, e.g., “down”, “bite”, “mommy”), 4. limited tongue tip elevation from jaw or limited posterior tongue movements, 5. inability to alternate place of articulation and/or planes of movement (e.g., lip rounding/retraction in “yoyo” or in multisyllabic words such as “ladybug”, “doubleyou”), 6. limited variety of speech movements (e.g., using jaw as primary articulator), 7. limited vowel and consonant repertoire and distortions of vowels and consonants, 8. limited syllable and word shapes, and 9. difficulty maintaining sound and syllable integrity with increased length and complexity of utterance^5^); and (h) demonstrated readiness for direct speech therapy and age-appropriate play skills. Due to slow participant recruitment, amendments (March 2014) to the inclusion criteria were made ~7.5 months following the start of the study/initial ethics approval (July 2013). These amendments pertain to an increase in the age range from 3–6 to 3–10 years old and the removal of restrictions in expressive language (from (f)). Children were excluded from the study if they presented with any of the following: (a) signs and symptoms suggesting global motor involvement (e.g., cerebral palsy); (b) more than 7 out of 12 indicators for CAS;^23^ (c) autism spectrum disorders; (d) oral structural/resonance issues; and (e) feeding/drooling issues.

### Intervention

Empirical studies to support the use of PROMPT for children and adults with speech disorders that affect speech motor planning and execution processes have been conducted, replicated, and validated by researchers and independent labs from around the world.^5,13,17,24,25^ In PROMPT intervention, goals are chosen to reflect the complex inter-relationships among physical-sensory, cognitive-linguistic, and social-emotional domains.^4^ A PROMPT clinician would use the motor-speech hierarchy (MSH^4^) to select speech motor goals for intervention. The MSH represents seven hierarchal and interactive developmental stages in speech motor control (stage I: tone; stage II: phonatory control; stage III: mandibular control; stage IV: labial–facial control; stage V: lingual control; stage VI: sequenced movements; stage VII: prosody). These hierarchical speech motor goals are embedded into cognitive-linguistic and social-emotional needs of the child. Intervention typically proceeds from the lowest subsystem in the MSH where a child has speech motor control issues. Specific techniques are used to stimulate sensory input (i.e., tactile, kinesthetic, proprioceptive, auditory, and visual) to facilitate the formation of sensory–motor pathways required for the acquisition and accurate production of speech movement patterns. Principles of motor learning,^26^ such as pre-practice considerations, practice schedules (e.g., blocked and random practice), knowledge of performance (KP; e.g., “use your small mouth”), and knowledge of results (KR; e.g., “that was very good”), were applied to intervention sessions depending on the child’s needs. Early in intervention, immediate, and frequent feedback (KR and KP) was provided after each speech production to facilitate acquisition of new speech motor patterns. As sessions progressed, feedback frequency was decreased to encourage the child to self-monitor and control their own speech output.^26^ Intervention was delivered by an experienced and PROMPT certified speech–language pathologist (SLP).

Caregivers of the children in the waitlist/home training control group received a four-page handout detailing speech, language, and literacy strategies to be carried out at home. The materials were developed primarily by the Erinoak Kids Center for Treatment and Development (https://www.erinoakkids.ca/home.aspx) and adapted from the literature.^27^ These handouts are provided as part of standard care in the province of Ontario, Canada to caregivers of children who are waiting to receive speech and language services. These strategies pertain to therapy readiness (e.g., follow your child’s lead, use simple language, get face to face), responding to a child’s effort to communicate (e.g., ask choice questions, repeat any part of the sentence that you understand, be a model for children to teach the skills of revision), and promoting early literacy skills (e.g., shared book reading, give lots of encouragement, explain book/print organization).

### Outcome measures

**Fig. 1:**
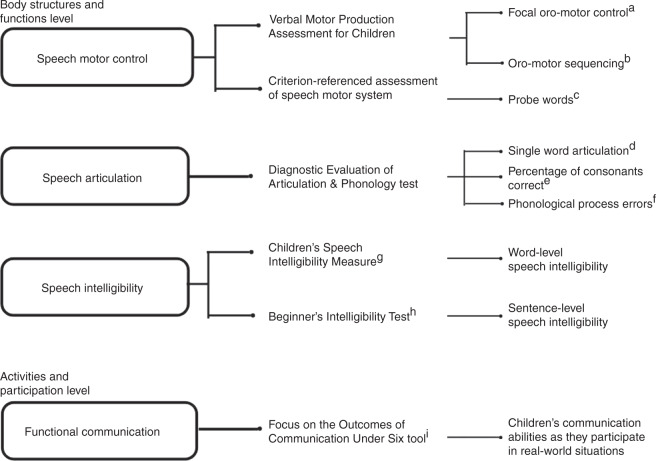
Outcome measures. Different outcome measures used to assess the body structures and functions, and activities and participation levels for the WHO-ICF-CY framework.^18^ See section on “Outcomes measures” for further details. **a** Verbal Motor Production Assessment for Children – Focal Oromotor Control.^28^ **b** Verbal Motor Production Assessment for Children – Sequencing.^28^ **c** Probe Words.^13^ **d** Single-word articulation assessment subtest of the Diagnostic Evaluation of Articulation & Phonology test (DEAP).^29^ **e** PCC, percentage of consonants correct derived from the DEAP test.^19^ **f** Single-word phonology assessment subtest of the DEAP.^29^ **g** Children’s Speech Intelligibility Measure (CSIM).^31^ **h** Beginner’s Intelligibility Test (BIT).^32^ **i** Focus on the Outcomes of Communication Under Six tool (FOCUS).^33^

Outcome measures were assessed at the body structures and functions level, and at the activities–participation level as per the WHO-ICF-CY^15,18^ framework (see Fig. 1). These outcome measures and reliability procedures were assessed by SLPs blind to both group and session (baseline or 10-week follow-up) allocation. All outcome measures were assessed at baseline and at a 10-week follow-up (i.e., 10 weeks of intervention or 10 weeks of waitlist/home training).

### Measures at body structures and functions level

- *Speech motor control* function was assessed using the Verbal Motor Production Assessment for Children (VMPAC^28^) and a criterion-referenced probe wordlist procedure.^13,24^ The VMPAC was standardized on typically developing children between the ages of 3 and 12 years. The VMPAC measures the accuracy and consistency of non-speech and speech productions on a three-point scale (0: incorrect; 1: partly incorrect; 2: correct), and aids the identification of the level of breakdown in motor-speech control in children. For the present study, two subsections of VMPAC were administered: (a) focal oromotor control (VMPAC-FOC): assesses jaw, lip, and tongue oromotor control in speech (e.g., “Say /a/”) and non-speech movements (e.g., “Show me how you bite”); and (b) sequencing (VMPAC-SEQ): assesses the ability to correctly produce speech and non-speech movements in sequential order (e.g., “Say /m-o-i, m-o-i, m-o-i, m-o-i/”). The obtained raw scores are divided by the sum of the corresponding subsection scores and converted to a percent score (min 0 to max 100). A criterion-referenced measurement of the speech motor system using Probe Words^13,24^ was also carried out. The probe wordlist consists of four levels with ten words at each level. These levels are based on the hierarchical development of the speech motor system:^4^ level 1 are words that focus on jaw-driven movements (e.g., “Bob”, “map”); level 2 are words that focus on labial–facial movements (e.g., “moon,” “feet”); level 3 are words that focus on lingual movements (e.g., “sun,” “dig”); and level 4 are words that capture sequences of movements that integrates jaw–lip–tongue movements (e.g., “banana,” “ice cream”). The probe word assessment uses a picture-naming task and the child’s responses are scored as 1 or 0 depending on whether they meet the appropriate movement criteria (e.g., jaw range, jaw stability, lip symmetry, lip rounding/retraction, voicing transition, labial–lingual transition). The scores are summed across all 40 words and can range from 0 to 298, with a higher score (at 10-week follow-up) implying better outcomes. For the present study, the scoring was conducted using recorded videos by blinded raters.
- *Speech articulation* was assessed at the single-word level using the Diagnostic Evaluation of Articulation & Phonology test (DEAP^29^). The DEAP test is a standardized, norm-referenced measure with published validity and reliability data for children between the ages of 3 and 8;11 years of age.^29^ Children are asked to name 30 pictures in the DEAP test, which evaluates a child’s ability to correctly articulate English vowels, diphthongs, and consonants in different word (initial, medial, and final) positions. Articulation errors are phoneme substitutions, omissions, distortions, and additions. Raw scores are calculated by summing the number of articulation errors across word positions (total possible articulation errors = 103). The DEAP test manual^29^ provides tables to convert raw scores to standard scores (*Z*) using the formula: *Z* = *X* − M/SD^30^ (where *X* is the raw score, M the mean, and SD the standard deviation). Standard scores use an equal interval scale and thus can be applied to track progress over time.^3,23^ The standard score ranges from 55 to 145, with a higher score at 10-week follow-up indicating better outcomes. PCC^19^ is calculated from the same 30-item picture-naming task as mentioned above for DEAP. PCC raw scores are obtained by dividing the number of consonant errors produced by the total number of consonants (67) in the syllable-initial and syllable-final positions. The resulting number is then multiplied by 100 to get a percent score (range 0–100), with a higher score implying a better outcome at 10-week follow-up. Phonological process errors are speech sound error patterns such as the substitution of a plosive consonant (e.g., /p, b, t, d/) for a fricative consonant (e.g., /s, f/) as in “soap” → “toap”, reducing the number of consonants in a consonant cluster (e.g., “snake” → “nake”) and consonant deletions (e.g., “cat” → “ca”). The DEAP manual evaluates ten such speech sound error patterns and the raw scores are calculated by adding the error patterns across 50 words (total possible speech sound error patterns = 214^29^). The raw scores are then converted to standard scores (ranging from 55 to 145) as described previously, where a higher standard score implies a better outcome following 10 weeks.
- *Speech intelligibility* was assessed using imitation tasks selected from a list of closed set of single words from the Children’s Speech Intelligibility Measure (CSIM^31^) and a list of open-set connected speech sentences from the Beginner’s Intelligibility Test (BIT^32^). A different list of single words and sentences were used for each child in the baseline and 10-week follow-up assessments. The child’s imitations of the clinician’s model were audio-taped and played to a group of three listeners who were blinded to both group and session (pre or post) allocation. No listener heard the same child or the list of words/sentences twice. In total, 135 listeners (age M = 22.61 years; SD = 3.94; 61% females) participated in the study. All listeners passed a hearing screen at 25 dB HL (hearing level) and reported little or no exposure to speech of children with speech disorders. Listeners were recruited from the University of Toronto.
  The CSIM is composed of 200 lists of 50 words. The children were required to imitate one randomly chosen wordlist. These words were played in random order to three naive listeners at a comfortable 70 dB SPL (sound pressure level) loudness level via headphones. The listeners’ task was to select (by circling) the word they think they heard from a list of 12 phonetically similar words and the word-level intelligibility score was calculated from the percentage of words correctly circled (min 0 to max 100).
  The BIT includes four lists of ten syntactically simple sentences that are four to six words in length. Each sentence is composed of one or two syllable words, which are familiar to children. Children’s sentence productions are played to listeners who are asked to write down what they thought the child said. Sentence-level intelligibility scores were calculated from the percentage of target words correctly transcribed (min 0 to max 100). Further details on the procedures for the validity, reliability, administration, and scoring of these tests are standardized and reported elsewhere in detail.^3,5,12,23,31,32^

### Activities and participation level

*Functional communication* was assessed using the Focus on the Outcomes of Communication Under Six tool (FOCUS^33^). FOCUS^33^ is a valid and reliable outcome tool that assesses the changes in children’s ability to communicate in everyday life and correlates well with quality-of-life measures.^33^ It is a standardized 50-item questionnaire that is rated on a seven-point rating scale by either a caregiver or a SLP. For the current study, we used the caregiver-scored questionnaire to assess changes in functional communication. The minimum and maximum scores that can be obtained on this measure are 0 and 350 points respectively, with a change of ≥16 points indicating a minimal clinically important difference (MCID^33^). A higher score at 10-week follow-up implies better outcomes.

### Sample size calculation

A priori power analysis was calculated based on effect sizes reported in an earlier study, which included 12 children (between the ages of 3;11 and 6;7 years) with moderate to profound SSD and motor-speech difficulties, who received PROMPT intervention for a duration of 9 weeks.^5^ Analysis indicated that between 5 and 22 participants per group were required for different variables (speech motor control, speech articulation, and speech intelligibility) to detect an intervention effect with a power of 0.95 and *α* level of 0.05. Sample size could not be calculated a priori for functional communication as no FOCUS data was available at the start of the study for the given intervention and/or population to inform sample size calculations. For the interim (or study mid-point) sample size calculation, CSIM and FOCUS were used. Sample sizes were calculated only for FOCUS and CSIM as there were no reported meaningful differences (cut-off scores) to consider for power analysis for other variables (e.g., speech motor control, articulation). For FOCUS, an SD of 67 (SD of waitlist/home training group) was chosen for the sample size calculation. Based on this SD, the required sample size was calculated to detect a MCID^33^ of 16 points with 80% power and two-sided *α* of 5%. This resulted in a sample estimate of 122 participants per group that was adjusted for measurement at two time points (correlation of 0.75 between pre/baseline and post) using an analysis of covariance (ANCOVA). For CSIM, an SD of 17 (of waitlist/home training group) was used for sample size calculations to detect a difference of 10% (absolute) with 80% power and two-sided *α* of 5%. These calculations yielded a sample estimate of 21 participants per group based on measurement at two time points (correlation of 0.75 between pre/baseline and post) for an ANCOVA analysis. However, a sample size of 122 per group is clinically and practically challenging to achieve in a reasonable timeframe for disorders with such low incidence/prevalence rates. Thus, we decided to terminate recruitment in the study with a clinically feasible final sample size of 25 participants per group based on CSIM sample size calculations, acknowledging the fact that results for the FOCUS outcome measure would be underpowered.

### Randomization

Randomization was conducted by an external data monitoring and safety agency, the AHRC at St. Michael’s Hospital in Toronto, Ontario, Canada. At AHRC, the randomization sequence was computer generated and stratified by clinical site with random permuted blocks of sizes 2 and 4. The randomized group allocations were placed in sequentially numbered, opaque, and sealed envelopes, which were provided to clinical sites at the start of the study by AHRC. Once the eligibility criteria were met and informed consent was obtained from each participant, the study coordinator at each clinical site opened one envelope that contained a group allocation. A new envelope was opened for every participant.

### Blinding

All study personnels (including the principal investigator, all research and clinical staff, and outcome assessors) were blind to group allocation, except for the study coordinator (at the local clinical site), the SLP delivering the intervention, and the participants. Outcome assessors (e.g., SLPs and listeners who evaluated speech intelligibility) received edited audio and video recordings of the child’s productions, which did not contain group/session allocation information.

### Data management

The ethics for this study was approved by the Research Ethics Board at the University of Toronto (Protocol #29142) and the study was registered at the Clinical Trials Registry prior to submission (#NCT02105402). The study’s integrity was maintained by AHRC who verified the consent process, conducted on-site data monitoring visits, ensured participants met the study inclusion/exclusion criteria, verified source data, conducted data entry verifications, randomized group allocation, and conducted interim power analysis and all statistical analysis on outcome measures. All consent forms and identifying information are stored in a locked filing cabinet at each respective clinical site and can only be accessed by the study coordinator and the AHRC. All experimental data were de-identified, blinded, and transferred to the research coordinating center at the Oral Dynamics Lab, University of Toronto. Digital (audio and video) files were stored and transferred on a Level 2 HIPAA Hardware Encrypted External Desktop Drive. All file storage and transfer met the requirements of the Personal Health Information Protection Act and ethics guidelines.

### Recording, fidelity, and reliability

All assessment and intervention sessions were video recorded (JVC Everio GZ-E220 HD: resolution 1920 × 1080) and audio recorded (using Zoom H1 Ver 2.0: resolution 16 bit/sample at 44.1 kHz) to calculate inter-rater reliability and intervention fidelity. *κ*-Statistics was used for inter-rater reliability calculations, where <0, 0.2, 0.4, 0.6, 0.8, and 1, are often referred to agreement that is poor, slight, fair, moderate, substantial, and almost perfect, respectively.^34^ The *κ* coefficient was calculated from 20% of all data by SLPs who were blind to group and session allocations. The average *κ* coefficient was 0.73 (substantial) for assessment transcriptions (International Phonetic Alphabet). The *κ* scores calculated from video recordings of Probe Words at the different levels ranged between fair and moderate: level 1, mandibular = 0.52; level 2, labial–facial = 0.57; level 3, lingual = 0.63; and level 4, sequenced = 0.48. To calculate internal consistency, the Cronbach’s *α* coefficient was used where <0.5, 0.6, 0.7, 0.8, and 0.9 are often referred to as unacceptable, poor, questionable, acceptable, good, and excellent, respectively for internal consistency levels.^35^ The Cronbach’s *α* scores calculated at the different levels of Probe Words ranged between acceptable and good: level 1, mandibular = 0.75; level 2, labial–facial = 0.79; level 3, lingual = 0.78; and level 4, sequenced = 0.83.

Intervention fidelity was calculated for 20% of all intervention sessions. Clinicians met the intervention fidelity requirement of >80% based on video recordings of intervention session using the fidelity checklist form.^16^ Audio recordings of the children’s productions of speech intelligibility items were saved as .wav files and played in random order to three naïve listeners using a headphone amplifier (PreSonus HP60) and headphones (Sony MDR-XD10) at 70 dB SPL.

### Statistical methods

All data entry verifications and primary statistical analyses were performed by a research biostatistician from the AHRC. All outcome measures were analyzed with an ANCOVA model with baseline scores set as a covariate using the intent-to-treat principle. Missing data were treated as missing in the analyses and presented in Table 2. Effect sizes were calculated from the regression models in the original units for each variable and reported along with their 95% confidence intervals. All statistical procedures were carried out using R software version 3.5.1^36^ with a two-sided *α* level of 0.05.

## Results

### Recruitment and participant flow

**Fig. 2:**
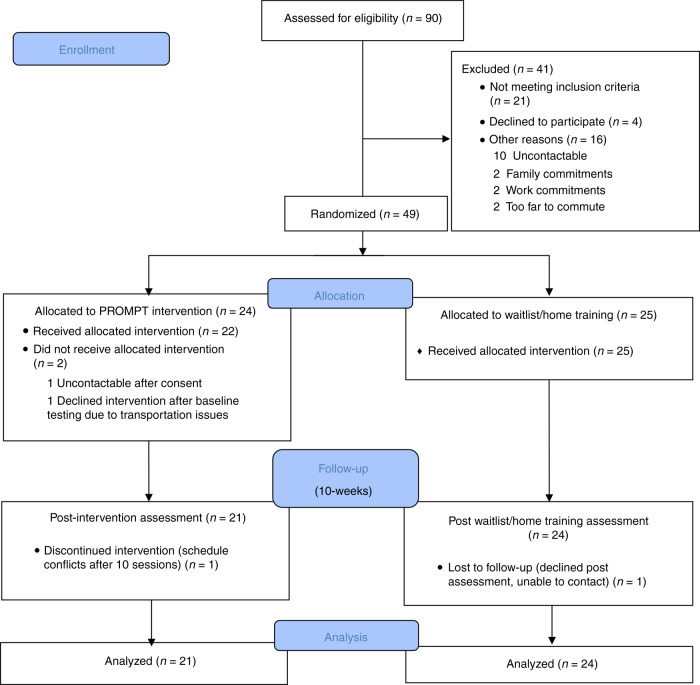
Consolidated Standards of Reporting Trials (CONSORT) flow diagram.

Recruitment and data collection for the study occurred between January 2014 and June 2017. Ninety children were assessed for eligibility and 49 children were randomized (*N* = 24 for the intervention group, mean age = 48.70 months, SD = 11.17; *N* = 25 for waitlist/home training group, mean age = 48.08 months, SD = 12.33). Forty-five children’s data were available for final analysis. The trial design and study flow is presented in Fig. 2 as per the Consolidated Standards of Reporting Trials (CONSORT^37^) guidelines. In the current study, all children met the age criteria for the standardized tests and outcome measures, except three children who were above the age of 6 years (6;1, 6;3, and 7;9 years) and did not align with the permissible testing age for the FOCUS measure.

### Baseline characteristics

Information on the study sample is presented in Table 1.

**Table 1:** Participant demographics (mean (SD); see section on “Participants and setting” for more details).

	PROMPT treatment	Waitlist/home training	Missing (%)
Participants	24	25	
Age (months)	48.70 (11.17)	48.08 (12.33)	2.0
Gender^a^			2.0
Female	9 (39.10)	10 (40.00)	
Male	14 (60.90)	15 (60.00)	
Primary language (English) spoken at home^a^	24 (100.0)	25 (100.0)	0.0
Hearing and vision (within normal limits)^a^	24 (100.0)	25 (100.0)	0.0
History of speech and language intervention^a^	13 (65.0)	19 (82.60)	12.2
P-TONI^b^			
Non-verbal index	103.95 (12.96)	106.39 (23.94)	12.2
PCC^c^	40.42 (15.22)	44.47 (19.15)	6.1
CELF^d^			
Receptive language index	91.81 (14.16)	101.12 (15.50)	32.7
Expressive language index	72.24 (11.53)	80.50 (18.34)	32.7
Mean number of indicators			
Motor-speech involvement^e^	8.11 (1.71)	8.29 (1.68)	20.4
Child apraxia of speech^f^	3.76 (0.83)	4.10 (1.02)	24.5

*SD* standard deviation, *P-TONI* Primary Test of Non-verbal Intelligence, *PCC* percentage of consonants correct, *CELF* Clinical Evaluation of Language Fundamentals, *DEAP* Diagnostic Evaluation of Articulation & Phonology test.

^a^Proportion of children.

^b^Standard scores of P-TONI.^20^

^c^Percentage of consonants correct extracted from the DEAP.^19,29^

^d^Standard scores of Clinical Evaluation of Language Fundamentals (CELF-4;^22^ CELF-P2^21^).

^e^Motor-Speech Involvement checklist.^5^

^f^Child Apraxia of Speech checklist.^23^

### Intervention delivery

PROMPT intervention was delivered using the following dose parameters: dose form (structured play in a quiet setting), dose (69.75 productions per goal), dose frequency (twice per week), session duration (45 min), total intervention duration (10 weeks), and Cumulative Intervention Intensity (1395 productions per goal). During the 10-week study period, caregivers of waitlist/home training control participants reported consistent use of strategies at home and none received any other speech–language interventions.

### Outcomes

Means, SDs (within parentheses), and missing data for all measures at baseline and 10-week follow-up are reported in Table 2. Table 3 represents effect sizes, 95% confidence intervals for effect sizes, and *p* values from ANCOVA model using the intent-to-treat principle with baseline as covariate. The ANCOVA analysis on 45 participants (PROMPT intervention group: *n* = 21; waitlist/home training group: *n* = 24) revealed that PROMPT intervention was associated with notable changes in speech motor control (VMPAC-FOC: *p* = 0.016, increase by 6.27%; Probe Words: *p* = 0.025; increase by 28.79 points), speech articulation (*p* = 0.002, standard score improvement of 5.15), PCC (*p* < 0.001, increase by 10.85%), and word-level speech intelligibility (*p* = 0.002, increase by 8.59%) relative to the waitlist/home training group. Intervention allocation yielded only weak differences in VMPAC-SEQ, phonological process errors, sentence-level speech intelligibility, and functional communication. As per CONSORT reporting guidelines, there were no changes to trial outcomes after the trial commenced.

**Table 2:** Descriptive statistics (mean (SD) and missing data for all measures at baseline and 10-week follow-up; see section on “Outcome Measures” for further details).

Outcome measures	PROMPT treatment (*n* = 24)		Waitlist/home training (*n* = 25)		Missing (%)	
	Baseline, mean (SD)	10-week Follow-up, mean (SD)	Baseline, mean (SD)	10-week Follow-up, mean (SD)	Baseline	10-week Follow-up
Speech motor control						
VMPAC-FOC^a^	68.57 (14.30)	78.55 (14.43)	66.26 (11.49)	69.11 (13.50)	6.1	14.3
VMPAC-SEQ^b^	51.58 (24.84)	61.09 (24.20)	47.19 (21.07)	52.93 (20.52)	6.1	14.3
Probe Words^c^	223.67 (80.82)	267.22 (57.94)	223.38 (75.42)	239.25 (69.79)	0.0	12.2
Speech articulation						
Single-word articulation^d^	61.82 (6.99)	67.50 (9.53)	64.40 (8.58)	65.83 (8.93)	4.1	10.2
PCC^e^	40.42 (15.22)	54.48 (17.36)	44.47 (19.15)	46.27 (20.02)	6.1	10.2
Phonological process errors^f^	59.76 (6.42)	62.00 (7.85)	65.21 (8.78)	64.35 (8.16)	8.2	12.2
Speech intelligibility						
Word level (CSIM)^g^	40.64 (15.07)	50.44 (16.97)	41.54 (16.30)	41.80 (14.59)	8.2	18.4
Sentence level (BIT)^h^	21.80 (19.41)	31.62 (23.41)	19.23 (15.01)	30.28 (19.87)	8.2	16.3
Functional communication						
FOCUS^i^	213.91 (54.26)	228.65 (52.99)	214.55 (56.81)	227.24 (53.93)	10.2	16.3

*DEAP* Diagnostic Evaluation of Articulation & Phonology test, *CSIM* Children’s Speech Intelligibility Measure, *BIT* Beginner’s Intelligibility Test.

^a^VMPAC-FOC, percentage scores from the Verbal Motor Production Assessment for Children – Focal Oromotor Control.^28^

^b^VMPAC-SEQ, percentage scores from the Verbal Motor Production Assessment for Children – Sequencing.^28^

^c^Probe Words, raw scores based on 40-word item list.^13^

^d^Standard scores derived from the single-word articulation assessment subtest of the DEAP.^29^

^e^PCC, percentage of consonants correct.^19^

^f^Standard scores derived from the single-word phonology assessment subtest of the DEAP.^29^

^g^Percentage scores from the CSIM.^31^

^h^Percentage scores from the BIT.^32^

^i^FOCUS, Focus on the Outcomes of Communication Under Six tool.^33^

**Table 3:** Outcome measure effect sizes, 95% CI of effect sizes, and *p* values (see section on “Outcomes” for further details).

Outcome measures	Effect size	Lower 95% CI	Upper 95% CI	*P* value
Speech motor control				
VMPAC-FOC^a^	6.270	1.223	11.318	0.016
VMPAC-SEQ^b^	4.769	−3.050	12.587	0.225
Probe Words^c^	28.790	3.748	53.832	0.025
Speech articulation				
Single-word articulation^d^	5.157	2.061	8.252	0.002
PCC^e^	10.855	6.166	15.545	<0.001
Phonological process errors^f^	1.858	−1.807	5.523	0.311
Speech intelligibility				
Word level (CSIM)^g^	8.595	3.283	13.907	0.002
Sentence level (BIT)^h^	−1.632	−11.059	7.796	0.728
Functional communication				
FOCUS^i^	2.042	−14.971	19.056	0.809

CI confidence intervals, DEAP Diagnostic Evaluation of Articulation & Phonology test, CSIM Children’s Speech Intelligibility Measure, BIT Beginner’s Intelligibility Test.

^a^VMPAC-FOC, percentage scores from the Verbal Motor Production Assessment for Children – Focal Oromotor Control.^28^

^b^VMPAC-SEQ, percentage scores from the Verbal Motor Production Assessment for Children – Sequencing.^28^

^c^Probe Words, raw scores based on 40-word item list.^13^

^d^Standard scores derived from the single-word articulation assessment subtest of the DEAP.^29^

^e^PCC, percentage of consonants correct.^19^

^f^Standard scores derived from the single-word phonology assessment subtest of the DEAP.^29^

^g^Percentage scores from the CSIM.^31^

^h^Percentage scores from the BIT.^32^

^i^FOCUS, Focus on the Outcomes of Communication Under Six tool.^33^

### Harms

No adverse events were identified at any point in the study.

## Discussion

To our knowledge, this is the first study to evaluate the effectiveness of PROMPT (2×/week, 10 weeks) intervention to improve the speech and functional outcomes in children with SMD relative to a waitlist/home training control. This study also represents the largest cohort of children with severe SSD assessed with a motor-speech intervention. The results indicate that a 10-week PROMPT intervention improved speech motor control, articulation, and word-level speech intelligibility in children with severe SMD. Although important effects were noted for these variables, intervention did not result in notable group differences for phonological process errors, oromotor sequencing, sentence-level speech intelligibility, and functional communication. As PROMPT is a motor-speech intervention, phonological process errors did not change as they were not targeted. It is interesting to note that changes at the level of speech motor control and word-level speech intelligibility do not translate to notable changes at longer units of production (e.g., sentence-level intelligibility) or to improvements in everyday functioning.^5^ It is likely that children with SMD with a pathophysiology at the level of neuromotor execution of speech^2^ are experiencing increased demands on their speech motor system during the production of longer and more complex utterances,^9^ and hence improvements in connected speech intelligibility may not be feasible in a short intervention period.^3,12^

In the current study, the effect sizes for focal oromotor control scores indicated a 6% increase. Clinically, for a 3-year-old child with SMD, this would imply that their speech motor skills are approaching the mean of typically developing children for that age group.^28^ For children over the age of 3, this 6% increase would represent a change from a severe to a mild deficit, indicating a clinically significant improvement.^28^ This implies that early intervention may facilitate the normalization of speech motor skills. For Probe Word scores, a 28-point change is approximately a 9% improvement in speech motor skills, which is similar to the change observed in other variables tested in the study and in the literature.^5,13^ Effect sizes for speech articulation indicate a five standard score change in the intervention group above and beyond maturation and home training, corresponding to a change from 0.4 to 2nd percentile rank. Generally, intervention services are provided to children who are below the 7th percentile.^38^ For PCC, a typical measure of speech severity, an effect size of 10% observed in this study indicated a clinically significant improvement in severity scores from severe to moderate–severe.^19,30^ Word-level speech intelligibility in the intervention group improved by ~8.5% in effect size relative to the control group. These values are comparable to those reported in the literature for similar populations.^3,12,31^ Further, at the end of 10 weeks of intervention, children in the study were ~31% intelligible at the sentence level, whereas typically developing children in this age range are expected to be between 71 and 99% intelligible.^39^ It is known that <60% intelligible speech negatively impacts a child’s interaction in social settings and should be considered as potential candidates for continued intervention to reach acceptable levels of intelligibility and functional communication.^40^

### Limitations

There were several limitations noted in the current study that may have influenced the outcome of the results. First, the current RCT requires high internal validity and was conducted under ideal conditions, including tightly controlled intervention dosage,^3^ and intervention provided by PROMPT Certified and Instructor-level SLPs with >10 years of experience. Future research should reflect real-world practice settings and adaptive variations in intervention delivery, as well as differences in SLP experience.

Second, although positive evidence of transfer and generalization of speech motor skills to articulation and ecologically valid speech intelligibility testing was noted, the study did not address maintenance of intervention effects. Maintenance of intervention gains over an extended period of time (e.g., 4 months) has been suggested to be a critical feature in differentiating intervention effectiveness for children with speech motor disorders.^8^ Follow-up studies on PROMPT intervention are encouraged to include maintenance testing in their study design.

Lastly, the lack of changes observed in the FOCUS outcome measure may reflect an inadequate sample size as noted earlier (see section on “Sample size calculation”). Despite conducting a multi-center RCT using active and elaborate recruitment procedures, it was clinically and logistically not feasible to recruit a large sample size for a disorder such as SMD that has low prevalence and incidence.^2^ Future research may consider budgeting for the recruitment of a larger sample size involving more sites.

## Conclusion

This is the first RCT to examine the efficacy of PROMPT intervention for children with severe SMD. The findings suggest that PROMPT intervention when provided for two times a week for 10 weeks results in notable improvements in speech motor control, articulation, and word-level speech intelligibility. However, this population may also need more than one 10-week block of therapy to reach acceptable levels of sentence-level intelligibility and functional communication. Overall, the findings suggest that PROMPT intervention is a clinically effective intervention approach for children with severe SMD.

## Data Availability

De-identified data can be obtained by emailing the corresponding author at a.namasivayam@utoronto.ca.
